# Local Anaesthetic Infiltration and Indwelling Postoperative Wound Catheters for Patients with Hip Fracture Reduce Death Rates and Length of Stay

**DOI:** 10.1155/2015/316817

**Published:** 2015-11-16

**Authors:** William D. Harrison, Deborah Lees, Jamie A'Court, Thomas Ankers, Ian Harper, Dominic Inman, Mike R. Reed

**Affiliations:** Orthopaedic Department, Wansbeck General Hospital, Northumbria Healthcare Trust, Woodhorn Lane, Ashington, Northumberland NE63 9JJ, UK

## Abstract

*Background*. An analgesic enhanced recovery (ER) protocol for patients with a hip fracture was introduced. It was hypothesised that the ER would reduce pain, length of stay and improve clinical outcomes. The protocol used intraoperative infiltration of levobupivacaine followed by ongoing wound infusions.* Methods.* Consecutive patients admitted to two hospitals were eligible for the ER protocol. Numerical Reporting Scale pain scores (0–10) were recorded alongside opiate requirements. 434 patients in the ER group (316 full ER, 90 partial ER, and 28 no ER) were compared to a control group (CG) of 100 consecutive patients managed with traditional opiate analgesia.* Results*. Mean opiate requirement was 49.2 mg (CG) versus 32.5 mg (ER). Pain scores were significantly reduced in the full ER group, *p* < 0.0001. Direct discharge home and mean acute inpatient stay were significantly reduced (*p* = 0.0031 and *p* < 0.0001, resp.). 30-day mortality was 15% (CG) versus 5.5% (ER), *p* = 0.0024.* Conclusions*. This analgesic ER protocol for patients with a hip fracture was safe and effective and was associated with reduced inpatient stay and mortality.

## 1. Introduction

Enhanced recovery initiatives for orthopaedic surgery have been shown to improve patient outcomes and effectively reduce service demand and costs [1–3]. Hip fracture is the most common trauma admission in the United Kingdom and is expected to become more common with an ageing population [4]. The general approach to hip fracture management has changed over the last decade with a drive to improve the associated morbidity and mortality. Recognising the multisystem needs of this at-risk patient group is crucial and is the focus of quality improvement programmes within the United Kingdom National Health Service.

Local infiltration of anaesthetic (LIA) intraoperatively and subsequent indwelling catheter infusion (CATH) for postoperative arthroplasty pain management are gaining popularity in the enhanced recovery setting. The combination of LIA and CATH is more accepted in total knee arthroplasty than in elective hip arthroplasty [5]. Results from studies looking specifically at LIA in hip arthroplasty have shown no clinical benefit compared to multimodal oral analgesia [6–9]. Early evidence from LIA and CATH for knee arthroplasty surgery demonstrates lower opiate requirements and overall pain scores when compared to intrathecal morphine [10]. LIA and CATH in conjunction with other pharmacological, procedural, and behavioral adaptations for an enhanced recovery protocol for knee arthroplasty demonstrated an increased patient satisfaction, reduced blood transfusions, reduced length of stay, and decreased mortality [3].

Level 1 evidence has demonstrated lower requirement for breakthrough opiates following LIA in hip arthroplasty [11, 12]. Busch et al. demonstrated a reduction of patient controlled opiate analgesia and reduced pain on activity, in their level 1 study of 64 patients [13]. However, there is conflicting evidence from other randomized studies that have demonstrated that LIA and particularly CATH have no short-term benefit in elective primary total hip replacement [5–7, 9]. The differences between the pain experienced after elective total hip replacement and the pain experienced after hip fracture fixation or hemiarthroplasty are speculative. Pain originating from the local damage to soft tissues and the fracture itself from trauma are more acute than the chronic pain experienced prior to elective arthroplasty surgery. Whether the nociceptive stimuli of trauma and arthritis respond differently to local anaesthetic infusions is not known.

Patients with hip fracture often have cognitive impairment and often receive an inequality of pain relief [14]. One aim of this analgesic enhanced recovery programme was to reduce this inequality and provide standard multimodal analgesia for all patients.

Other benefits of LIA and CATH are to reduce the volume of opiates and subsequently reduce the adverse side effects [12, 15, 16]. Opiates are renally excreted and the elderly are at risk of significant opiate sensitivity leading to respiratory depression, hypoxia, lower respiratory tract infection, delirium, and constipation. There are however potential risks of using high doses of local anaesthetic in this frail elderly group with possible systemic local anesthetic toxicity with central nervous or cardiorespiratory compromise [2, 17, 18].

The aim of this study is to establish if local infiltration of anaesthetic (LIA) and indwelling anaesthetic catheter infusions (CATH) are superior to standard analgesia used in a control group (CG) for management of patients with a hip fracture.

## 2. Method

This is a study of consecutive patients presenting to two separate acute hospitals between April 2010 and May 2012. Wansbeck General Hospital (hospital 1) and North Tyneside General Hospital (hospital 2) are both governed under the Northumbria Healthcare Trust and are 15 miles apart. Enhanced recovery protocols for hip fractures are a multimodal optimisation of patient care from all facets including nutrition, physiotherapy, timely surgery, and perioperative analgesia. This study looks at a single facet of care, namely, analgesia. For the sake of simplicity, the term enhanced recovery (ER) is used to describe the full analgesic protocol offered to hip fracture patients and no other interventions. We therefore acknowledge that this is not a complete enhanced recovery protocol in the full sense of the term.

The aim was to gain an accurate representation of how the ER protocol impacts on pain scores, opiate requirements, and outcomes following hip fracture. Local Caldicott approval was obtained. There are two arms to this retrospective study: control group (CG) and the ER.

It was recognised that not all patients with a hip fracture could be treated with the full ER protocol. Components and variations of the enhanced recovery (ER) protocol include the following:

1. The full ER protocol:
  - including both LIA and CATH together.
2. CATH only (analysed as a subgroup) due to
  - anaesthetist preference;
  - recent local anaesthetic nerve block given instead.
3. LIA only (analysed as a subgroup) due to
  - risk of cumulative local anesthetic toxicity;
  - agitated patients at risk of pulling out CATH.
4. Non-ER protocol group receiving no aspects of the ER due to
  - a risk of local anaesthetic toxicity and risk of pulling out CATH;
  - these patients who were managed with only traditional oral and parenteral analgesia as an alternative;
  - non-ER protocol patients receiving the same analgesia as the CG, but as they were treated at the time of the ER protocol they were not consecutive or unselected.

The CG group includes 100 consecutive patients treated immediately before the introduction of ER in April 2010. They received oral and parenteral multimodal analgesia only. Fifty consecutive patients from each of the two recruiting hospitals were selected.

Both the CG and the ER had the same protocol for admission fast-tracking to a trauma ward and were prioritised for theatre within 36 hours. Orthogeriatric input was mandatory within the first 24 hours of admission between both groups. A formal analgesia, laxative, and antiemetic protocol was equivalent between both groups. There was a large crossover in the rehabilitation facilities available to both hospitals due to their proximities. Discharge criteria were multifactorial but consistent between both hospitals during the ER and CG. Discharge was dictated by a consultant assessment of medical fitness, occupational therapy assessment of social circumstances, and physiotherapy assessment of mobility. One difference between the groups was the employment of a dedicated nutritionist for hip fracture patients in the latter half of the ER protocol data collection at both hospitals.

In both groups patients managed nonoperatively were excluded.

Data was gathered from medical notes, physiotherapy notes, medication charts, observation charts, and theatre records. Patient demographics, comorbidities, fracture pattern, and type of operative management were recorded.

Pain scores were measured according to the Numerical Rating Scale (NRS) between 0 and 10 [19]. The NRS scores were documented by nursing staff on each occasion of recording of postoperative observations. Patients with cognitive impairment who could not provide NRS for pain did not have a value recorded and were not included in the analysis of the NRS. The development of new confusion in the postoperative period was documented as it may also interfere with the quality NRS pain scores. In addition to pain scores, it was recognised that confused patients have more complex social needs, often delaying their discharge. Therefore a separate subgroup of patients without cognitive impairment were analysed to define the impact of the ER protocol on their discharge outcomes.

Nursing staff and patients were not blinded to those who received ER. Nurses provided analgesia for all patients requiring breakthrough pain relief regardless of the new ER protocol. Postoperative analgesia requirement for all patients was recorded, including “regular” and “as required” analgesia. All multimodal postoperative analgesia was recorded including paracetamol, mild opiates, and morphine.

The destination of discharge, that is, own home, residential care, or nursing home, was recorded for each patient. Direct discharge to the patients' home was considered the major endpoint in care within patient mortality and discharge to another care facility affecting this endpoint. The length of stay (on an acute ward) was also an important outcome measure. The duration of care in the rehabilitation facility was recorded when applicable. Thirty-day mortality of patients during the acute hospital admission was recorded for all patients. Data collection was undertaken exclusively by the authors.

Statistical analysis was performed using GraphPad Prism version 5.3 using the one-way ANOVA test to delineate between CG, LIA only, CATH only, and the “ER” (both LIA and CATH together). Fisher's exact test (two-tailed) was used for two-sided outcome analysis.

### 2.1. Enhanced Recovery (ER) Technique

Levobupivacaine (0.125%, 100 mL) (Chirocaine, Abbott Laboratories, Illinois, USA) was infiltrated (LIA) intraoperatively in a wide and layered field including joint capsule, muscle, fat, and skin. An epidural catheter (CATH) was positioned with the tip of the catheter deep to the joint capsule for an arthroplasty procedure and deep to the fascia lata for a fixation procedure. This CATH has a microbiological filter and exits away from the surgical field. 20 mL of levobupivacaine was infused through the catheter after skin closure and also for postoperative boluses (at 6, 14, and 24 hours). The AmbIT pump (Summit Medical Products, Inc., Sandy, UT) was used to deliver the boluses and the theatre and ward nursing staff received regular sessions to train and update them in using this device. After the fourth bolus, local anaesthetic was discontinued and the catheter was removed on the ward.

## 3. Results

There were 434 patients recorded during the ER period. Exclusions for nonoperative management were 2% (*n* = 2) during the CG period and 1.6% (*n* = 7) of patients during the ER period (see Table 1).

The outcomes of patients in the CG are compared to those in the ER period in Table 2. The decrease in 30-day mortality was significant (Fisher's exact test, *p* = 0.0024). Length of stay decreased from 15 days (CG) to 10 days (ER); however the proportion of patients being transferred to rehabilitation facilities increased, *p* < 0.0001. On subgroup analysis of cognitively intact patients only (Table 3), significantly less CG were discharged to their own home compared to the ER (*p* = 0.0031) and reduced the requirement for further nursing care (36.7% for CG versus 27% for ER, *p* = 0.1159). All cognitively intact patients received some form of the ER protocol. Inpatient confusion was matched between the CG and the enhanced recovery group (*p* = 0.087).

Comparing all cognitively intact patients in the CG with those in the enhanced recovery period also demonstrates a decrease in 30-day mortality, a decrease in direct discharge home, and an increase in discharge to another care facility.

**Figure 1:**
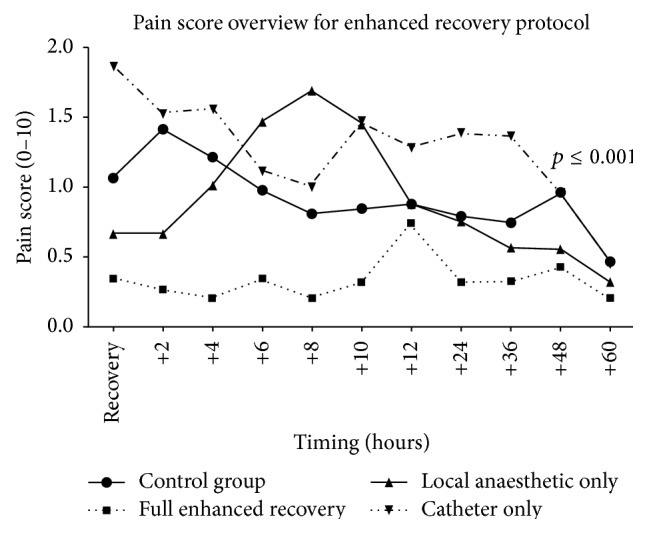
Numerical Reporting Scale (NRS) pain scores for the control group and the enhanced recovery subgroups.

Figure 1 demonstrates that cognitively intact patients in the CG reported significantly higher pain over the first 3 days compared to the cognitively intact members of the full ER, *p* < 0.0001. The LIA initially reported low pains scores compared to the CG, but by 6 hours NRS pain scores were higher in the LIA. Patients with CATH had a higher level of pain compared to the CG despite equivalent regular and as required analgesia. Patterns of NRS pain scores in the subgroups of the enhanced recovery protocol matched the patterns in opiate requirement, as seen in Table 4.

There were no identified episodes of local anaesthetic toxicity in all 406 patients who received levobupivacaine via LIA and/or CATH.

Data on superficial and deep infection was not routinely recorded in our database and therefore a breakdown of infection rates for each subgroup of the ER is not available. However, data submitted to Public Health England for the Surgical Site Infection Surveillance Service was available for review for each hospital, specifically for “repair of hip fracture” and with a quarterly breakdown of cases. Within the 3 months of the CG data collection, there were zero superficial infections and one episode of deep infection (0.3% of cases). During the ER protocol in 2010 and 2011, there were no superficial infections. Over the 48 months of the ER protocol data collection, there were six deep infections in hospital 1 (0.9% of cases versus national average of 1.7% of cases) and nine deep infections in hospital 2 (1.5% versus national average of 1.7% of cases) (Public Health England for the Surgical Site Infection Surveillance Service for “Repair Neck of Femur”).

Nursing staff did comment that cognitively impaired patients occasionally picked at the CATH dressing; however only 1.4% (*n* = 6) of patients had a catheter that was recorded as being removed prematurely. Similarly, 1.8% (*n* = 8) of patients with the CATH experienced intraluminal blockage of the catheter.

## 4. Discussion

This study has been performed over a 2-year period, in two hospitals, utilising a control group. The 100 patients retrospectively selected for the control group were identified as the most recently treated patients prior to the ER protocol in the two hospitals. This paper illustrates a working and practical model of an enhanced recovery protocol for patients with hip fractures.

The impact on the duration of inpatient stay was striking. The ER protocol provided a 5-day reduction in total length of stay (*p* < 0.0001) (Table 2). Although the discharge policy did not formally change between the CG and the ER, there may be other confounding factors which impact on this reduction. The reduction in acute stay did not extend into the mean duration of stay in further care, with both the CG and the ER having a mean stay of 15 days in rehabilitation. The reduction of 30-day mortality may have influenced the length of stay of survivors and the significant increase of patient discharges to rehabilitation hospitals during the enhanced recovery period, 12% (CG) versus 31.8% (enhanced recovery period), Fishers exact test, *p* < 0.0001.

There were 24 deaths during the data collection of the ER protocol period, including 14 patients who did not receive LIA or CATH. There were no inpatient deaths in the 230 patients who underwent the full ER protocol. There was a significant overall reduction of 30-day mortality from 15% (CG) to 5.5% (enhanced recovery period), *p* = 0.0024 (Fisher's exact test).

The lack of deaths in patients with the full ER protocol is striking. This may simply relate to pain relief and reduced opiate use. However, the use of continuous local anaesthetic has been shown to reduce postoperative ileus [20, 21], postoperative neurocognitive decline [22], and acute lung injury [23]. There is also evidence that local anesthetic has antimicrobial properties, particularly against* Staphylococcus aureus, Enterococcus faecalis, and Escherichia coli* in wound infections [24]. The Public Health England data on local infection rates is reassuring as no excess of deep infection was attributable to the ER period. In fact, the superficial and deep infection rates for hip fracture surgery remained lower than the national average.

The use of the NRS pain scores allowed efficient data collection of a large number of patients over many different measuring points during the inpatient stay. The disadvantage is that the 40.1% (*n* = 177) of patients with cognitive impairment in the enhanced recovery period could not report an objective score of their pain. Furthermore, pain and inadequate analgesia contribute towards confusion in elderly patients [25, 26] and previous studies have shown that the local anaesthetic blocks can reduce the prevalence of delirium in hip fracture patients [27, 28]. Tools exist for identifying pain levels in patients with cognitive impairment; however these are time-consuming and subjective and require a great deal of experience for the assessor [29]. The NRS pain scores in patients with normal levels of cognition were significantly lower throughout the hospital stay in the ER group than in the CG group (one-way ANOVA test, *p* < 0.001). The authors recognise that although the reduction of pain is statistically significant, it is only a reduction in the range of 0.5–1 out of 10 (Figure 1). This small change questions the clinical importance of this pain reduction; however, the combination of the demonstrable reduction in opiate requirement would support the effectiveness of the analgesic effect.

The provision of analgesia in the patients with dementia is an important humanitarian issue. Patients with cognitive impairment are often overlooked for opiate analgesia due to fluctuating consciousness and they do not express their severity of pain by the usual means [14]. The enhanced recovery protocol removes this discrimination and allows patients with cognitive impairment adequate and continued analgesia.

By using the LIA in isolation there was a clear reduction in pain over the first 4 hours, at which point there was a rise in postoperative pain (Figure 1). After this rise, the pain scores became more consistent with patients in the CG. The pattern may illustrate the half-life of the levobupivacaine at this site (approximately 2 to 2.6 hours). Despite the pattern in Figure 1, LIA has been shown to reduce wound pain sensitivity for up to three months following elective surgery [16]. In a study of 300 randomly assigned hip arthroplasty patients it is hypothesised that lower levels of acute postoperative pain impact on lower chronic pain experienced by the patient [30]. The analysis of the 15 patients (3.5% of the enhanced recovery period) who were managed with the CATH only reported a higher level of postoperative pain and however utilised less opiates than the CG. The CATH patients had a relatively high morphine requirement of 46 mg versus 31.8 mg in the ER group and also had a high mortality rate of 4 out of 15 patients (28.6%). The 30-day mortality figures in the CATH group may represent high-risk patients in whom the anaesthetist had deemed it unsafe to administer LIA.

The impact on opiate analgesia was evident in the ER group and to a lesser extent in LIA and CATH in isolation (Table 4). The reduction of morphine intake in this elderly group is important in order to decrease the potentially severe consequences of opiate toxicity. In terms of overall patient outcomes, the reduction of morphine intake may have contributed to the reduced mortality rate.

This study has limitations. Three-quarters (75.1%) of those in the ER group also received fascia iliaca compartment block (FICB) in the emergency department on admission to hospital. The FICB technique was gaining popularity in the emergency department during the time of data collection for the ER protocol. FICB was an analgesic intervention which aimed to supplement preoperative pain relief and was not considered as part of the postoperative pain relief delivered by the LIA and CATH. In 27 patients, surgery was prompt enough after a FICB that the full ER package was not delivered, to avoid local anaesthetic toxicity. There were no patients in the CG who received the FICB. As the half-life of levobupivacaine is 2–2.6 hours [31], any patient who receives a FICB and is operated on within 12 hours may receive local anaesthetic that is in addition to the ER. The FICB is therefore a confounding factor in this paper. Another limitation is that the CG and ER may not be comparable groups based on variations in treatment allocation and discharge destination. Randomising treatment groups would have provided clarity on this matter and given a clearer understanding of the impact of LIA and CATH on final outcomes. Nursing staff delivering postoperative analgesia and recording NRS pain scores were not blinded, as they needed to deliver the CATH analgesia on the wards. This may contribute to a study effect bias, as those in the ER may have been deemed not to require additional oral analgesia. However, nursing staff were encouraged to provide analgesia on an individual need basis.

Previous studies have commented on the cost effectiveness of LIA and CATH stating that the protocol is too expensive to justify in elective cases [32]. A cost analysis of local anaesthetic used in an enhanced recovery protocol for elective joint arthroplasty is awaiting publication [30]. The cost of the full ER is estimated at £138 per patient, with a breakdown of consumables of levobupivacaine at £24, the catheter at £8, and the AmbIT pump at £30 each [33]. Jones reported elective orthopaedic bed costs of £285 per day in 2008 [34]. According to the mean reduction in acute length of stay between the enhanced recovery period and the CG (5.1 days), the estimated saving per person is £1315.50. Excluding patients with cognitive impairment, those who received ER were also more likely to return directly home (*p* = 0.0031) rather than a care facility (Table 3). This may have far-reaching cost benefits for the healthcare system that surpass the short-term costs of staff training and equipment.

This study demonstrates the effective use of an enhanced recovery programme applied to patients with a hip fracture. Local anaesthetic as part of an enhanced recovery programme for patients with a hip fracture is favorable to traditional opiate-centered analgesia in terms of pain relief, duration of inpatient stay, discharge directly to home, and 30-day mortality. Patients receiving local anaesthetic infiltration and delivery by catheter have a better outcome than either technique in isolation. No patients receiving an intra-articular catheter developed deep wound infection and there were no recorded episodes of local anaesthetic toxicity. This enhanced recovery protocol can be considered to be a safe way to improve patient outcomes. A randomised controlled trial should be undertaken and specific attention should be made to the impact on mobility, morbidity, and 30-day mortality.

## Figures and Tables

**Table 1:** Patient demographics, mechanism of injury, fracture pattern, and management.

	Control group	Enhanced recovery period
*N*	100	434
Age mean	78.5 years	82.2 years
(Range)	(45–99)	(44–100)
Gender		
Male	24 (24.0%)	108 (24.8%)
Female	76 (76.0%)	326 (75.2%)
Inpatient confusion	31 (31.0%)	177 (40.1%)
Injury details		
Slip/trip	43 (43.0%)	191 (44%)
Collapse	4 (4.0%)	25 (5.8%)
Activity related	3 (3.0%)	49 (11.3%)
Slip on ice	0 (0.0%)	8 (1.8%)
Intoxicated	1 (1.0%)	7 (1.6%)
Fall in hospital	4 (4.0%)	9 (2.1%)
Assaulted	1 (1.0%)	0 (0%)
Unknown	41 (41.0%)	132 (30.4%)
Pathological	3 (3.0%)	13 (3.0%)
Fracture type		
Intracapsular	59 (59.0%)	239 (55.1%)
Extracapsular	35 (35.0%)	133 (30.6%)
Basicervical	4 (4.0%)	42 (9.7%)
Pertrochanteric	0 (0.0%)	14 (3.2%)
Subtrochanteric	2 (2.0%)	5 (1.2%)
Greater trochanter	0 (0.0%)	1 (0.2%)
Side		
Bilateral	0 (0.0%)	1 (0.2%)
Left	48 (48.0%)	217 (50%)
Right	52 (52.0%)	216 (49.8%)
FICB given?		
Yes	NA	326 (75.1%)
No	NA	108 (24.9%)
Procedure		
Nonoperative	2 (2.0%)	7 (1.6%)
Cannulated screws	7 (7.0%)	20 (4.6%)
Dynamic hip screw	26 (26.0%)	144 (33.2%)
Intramedullary fixation	16 (16.0%)	17 (3.9%)
Cemented bipolar hemiarthroplasty	2 (2.0%)	3 (0.7%)
Cemented Exeter hemiarthroplasty	11 (11.0%)	49 (11.3%)
Cemented Thompson's hemiarthroplasty	29 (29.0%)	183 (42.2%)
Uncemented Austin Moore hemiarthroplasty	1 (1.0%)	0 (0%)
Cemented Austin Moore hemiarthroplasty	3 (3.0%)	0 (0%)
Uncemented THR	1 (1.0%)	0 (0%)
Cemented THR	2 (2.0%)	15 (3.5%)
Enhanced recovery		
None	100 (100%)	28 (6.5%)
Full ER	NA	316 (72.8%)
LIA only	NA	75 (17.3%)
CATH only	NA	15 (3.5%)
Reasons for partial/no enhanced recovery		
Total	NA	118 (27.2%)
Renal impairment	NA	44 (10.1%)
Catheter pulled out by patient	NA	6 (1.4%)
Catheter blocked	NA	8 (1.8%)
Femoral nerve block given	NA	27 (6.2%)
Reason not documented	NA	31 (7.1%)
Previous adverse drug reaction to local anaesthetic	NA	2 (0.4%)

**Table 2:** Patient outcomes for the control group and the enhanced recovery cohort.

	Control group	Enhanced recovery cohort
*N*	100	434
Length of stay, mean (range)		
Orthopaedic ward	15 (3–114) days	10 (3–44) days
Rehabilitation	15 (1–64) days	15 (1–114) days
Sum duration for a 100-patient group	1680 days	1470 days
Discharge destination		
30-day mortality	15 (15%)	24 (5.5%) *p* = 0.0024
Own home	52 (52%)	162 (37.3%) *p* = 0.0090
Rehabilitation	12 (12%)	138 (31.8%) *p* < 0.0001
Care home	21 (21%)	110 (25.3%) *p* = 0.4393

**Table 3:** Subgroup analysis of management options and patient outcomes. Patients with cognitive impairment are excluded.

	Control group	Enhanced recovery period		
		Full ER	LIA only	CATH only
*N*	79	230	49	15
Discharge destination				
30-day mortality	7 (8.8%)	0 (0%)	3 (6.1%)	4 (26.7%)
Own home	43 (54.4%)	168 (73%)	31 (63.3%)	2 (13.3%)
Care facility	29 (36.7%)	62 (27%)	15 (30.6%)	9 (60%)
Length of stay in days				
Ortho. ward (mean)	15	9	9	10
Rehab (mean)	19	17	17	18
Total	34 (3–114)	26 (3–80)	26 (3–88)	28 (3–82)

**Table 4:** Opiate requirement for the control group versus the enhanced recovery protocol. Patients with cognitive impairment are excluded.

	Control group	Enhanced recovery period		
		ER	LIA only	CATH only
*N*	100	230	49	15
Cumulative mean	49.2 mg	31.8 mg	37.8 mg	46 mg
(Range)	(0–80 mg)	(0–98 mg)	(0–82 mg)	(3–55 mg)
